# Mono- or Double-Site Phosphorylation Distinctly Regulates the Proapoptotic Function of Bax

**DOI:** 10.1371/journal.pone.0013393

**Published:** 2010-10-14

**Authors:** Qinhong Wang, Shi-Yong Sun, Fadlo Khuri, Walter J. Curran, Xingming Deng

**Affiliations:** 1 Department of Pharmacology and Cancer Biology, Duke University Medical Center, Durham, North Carolina, United States of America; 2 Department of Hematology and Medical Oncology, Emory University School of Medicine and Winship Cancer Institute, Emory University, Atlanta, Georgia, United States of America; 3 Department of Radiation Oncology, Emory University School of Medicine and Winship Cancer Institute, Emory University, Atlanta, Georgia, United States of America; Calypte Biomedical Corporation, United States of America

## Abstract

Bax is the major multidomain proapoptotic molecule that is required for apoptosis. It has been reported that phosphorylation of Bax at serine(S) 163 or S184 activates or inactivates its proapoptotic function, respectively. To uncover the mechanism(s) by which phosphorylation regulates the proapoptotic function of Bax, a series of serine (S)→ alanine/glutamate (A/E) *Bax* mutants, including S163A, S184A, S163E, S184E, S163E/S184A (EA), S163A/S184E (AE), S163A/S184A (AA) and S163E/S184E (EE), were created to abrogate or mimic, respectively, either single or double-site phosphorylation. The compound *Bax* mutants (*i.e*. EA and AE) can flesh out the functional contribution of individual phosphorylation site(s). WT and each of these Bax mutants were overexpressed in Bax^−/−^ MEF or lung cancer H157 cells and the proapoptotic activities were compared. Intriguingly, expression of any of Bax mutants containing the mutation S→A at S184 (*i.e*. S184A, EA or AA) represents more potent proapoptotic activity as compared to WT Bax in association with increased 6A7 epitope conformational change, mitochondrial localization/insertion and prolonged half-life. In contrast, all Bax mutants containing the mutation S→E at S184 (*i.e*. S184E, AE or EE) have a mobility-shift and fail to insert into mitochondrial membranes with decreased protein stability and less apoptotic activity. Unexpectedly, mutation either S→A or S→E at S163 site does not significantly affect the proapoptotic activity of Bax. These findings indicate that S184 but not S163 is the major phosphorylation site for functional regulation of Bax's activity. Therefore, manipulation of the phosphorylation status of Bax at S184 may represent a novel strategy for cancer treatment.

## Introduction

Programmed cell death/apoptosis plays a critical role in normal development and maintenance of tissue homeostasis. This important cellular apoptotic process eliminating unwanted or damaged cells that prevents cancer development is mainly activated through intrinsic and extrinsic apoptotic pathways [1]–[2]. The extrinsic pathway is mediated by cell surface death receptor, such as TNF alpha receptor 1 (TNFR1), CD95/Fas and TRAIL, which activates caspase-8 followed by caspase-3 activation, while intrinsic pathway is characterized by mitochondrial dysfunction and activation of caspase 9 and Apaf-1 by cytochrome c (Cyt c) and other proteins including apoptosis inducing factor (AIF), Smac/Diablo and Endo G [1], [3], [4]. Bcl2 family members play a vital role in intrinsic apoptotic pathway by controlling outer mitochondrial membrane permeabilization (OMMP) [3], [5]. While the anti-apoptotic proteins, such as Bcl2, Bcl-XL and Mcl-1, prevent the release of Cyt c and AIF from mitochondria, the pro-apoptotic proteins, which are divided into two groups, including the multidomain-BH1-3 proteins (*i.e.* Bax and Bak) and the BH3-only proteins (*i.e*. Bid, Bim, Bik, Bid and Puma), facilitate apoptosis. Bax and Bak have been shown to be required for the intrinsic apoptotic pathway [6]. Bax is normally distributed in cytoplasm or loosely associated with mitochondrial membrane, whereas Bak is tightly associated with mitochondrial membrane [7]–[9]. Upon stimulation of apoptosis, Bax can be induced to undergo conformational change followed by formation of membrane-inserted homo-oligomers that result in OMMP [5], [10]. The anti-apoptotic proteins, such as Bcl2 and Bcl-XL, inhibit Bax conformational change and insertion into outer mitochondrial membrane, whereas BH3-only proteins activate Bax by binding to and antagonizing anti-apoptotic proteins or directly binding to and activating Bax [11]–[14]. Besides anti-apoptotic proteins of Bcl2 family and BH3-only proteins of this family, several non-Bcl2-related proteins, including Bif-1 [15]–[16], 14-3-3 [17], humanin (HN) peptide [18] and p53 [19]–[21], have also been reported to be involved in regulation of Bax activity. Bif-1 binds to and activates Bax, and p53 was shown to translocate from nucleus to mitochondria to induce Bax activation and Cyt c release [19], whereas14-3-3 and HN peptide inhibit Bax activity via direct interaction with this pro-apoptotic protein in cells [17]–[18].

Phosphorylation has been demonstrated to functionally regulate most of Bcl2 family members. For instance, phosphorylation of Bcl2 has been reported to enhance its anti-apoptotic capability [22], whereas serine (S) phosphorylation of the pro-apoptotic protein Bad inhibits its pro-apoptotic activity [23]. It has been reported that activation of AKT blocks conformational change of Bax as well as its translocation to mitochondria under stress stimuli [24]. The residue S184 in the C-terminus of Bax is a major site that can be phosphorylated in neutrophils in an AKT-dependent manner and phosphorylation at S184 is essential for the cytosolic retention of Bax [25]. In support of these findings, we recently discovered that nicotine can stimulate Bax phosphorylation at S184 through activation of AKT in association with increased survival and chemoresistance of human lung cancer cells [26]. Inversely, PP2A-mediated dephosphorylation of Bax at S184 enhances the proapoptotic function of Bax [27]. Another phosphorylation site of Bax is S163 that has been shown to be phosphorylated by GSK-3β *in vitro and in vivo*, which is associated with promotion of apoptosis in neuronal cells [28]. Moreover, it has recently been demonstrated that Bax can also be phosphorylated at threonine(T)167 site by stress-activated JNK and/or p38 kinase, which leads to Bax activation and mitochondrial localization prior to apoptosis [29].

The proapoptotic activity of Bax can be regulated by phosphorylation but the molecular mechanism(s) involved is not fully understood. The purpose of this study is to investigate the mechanism of how phosphorylation status of Bax at distinct site(s) affects the Bax-mediated mitochondrial cell death pathway. Here we chose the site-directed mutagenesis approach to generate the non-phosphorylatable and the phosphomimetic Bax mutants at different site(s). Results indicate that the S184 phosphorylation site plays a more important role than the S163 phosphorylation site in regulating the proapoptotic function of Bax.

## Materials and Methods

### Materials

Bax, Cyt c, prohibitin and caspase 3 antibodies were obtained from Santa Cruz Biotechnology (Santa Cruz, CA). T7 tag antibody was purchased from Novagen (San Diego, CA). Dual luciferase assay kit was purchased from Promega (Madison, WI). MitoTracker Red CM-H_2_XRos was purchased from Invitrogen-Molecular Probes (Carlsbad, CA). 6A7 antibody was obtained from BD PharMingen (San Diego, CA). Cyclohexamide was purchased from Sigma (St. Louis, MO). The site-directed mutagenesis kit was obtained from Clontech (Palo Alto, CA). Mitochondrial membrane potential detection kit was obtained from Cell Technology, Inc. (Mountain View, CA). All other reagents used were obtained from commercial sources unless otherwise stated.

### Plasmids, cDNA, cell lines and transfections

A series of the nonphosphorylatable and phosphomimetic at single site (*i.e*. S163 or S184) or the compound *Bax* mutants, including S163A, S184A, S163E, S184E, S163E/S184A (EA), S163A/S184E (AE), S163A/S184A (AA) and S163E/S184E (EE), were created as described previously [22]. The 5′ phosphorylated mutagenic primers for mutating S163 or S184 were synthesized as follow: S163A, 5′GAA GGC CTC CTC GCC TAC TTC GGG ACC 3′; S163E, 5′ GAA GGC CTC CTC GAG TAC TTC GGG ACC 3′; S184A, GTC CTC ACC GCC GCG CTC ACC ATC TGG 3′; S184E, GTC CTC ACC GCC GAG CTC ACC ATC TGG 3′. The T7-tagged WT-*Bax*/pUC19 construct was used as the target plasmid which contains a unique NdeI restriction site for selection against the unmutated plasmid. The NdeI selection primer is: 5′ GAG TGC ACC ATG GGC GGT GTG AAA 3′. Nucleotides corresponding to each serine (S) residue were substituted to create a conservative alteration to alanine (A) or glutamic acid (E) with a site-directed mutagenesis kit (Clontech) according to the manufacturer's instructions. Based on individual site *Bax* mutant(s), the compound *Bax* mutants were further created using a similar approach. Each single mutant was confirmed by sequencing of the cDNA and was then cloned into the pCIneo (Promega) mammalian expression vector. The pCIneo plasmid containing each *Bax* mutant cDNA was transfected into Bax^−/−^ MEF cells (a kind gift from Dr. Douglas R. Green, St Jude Childrens Research Hospital, Memphis, Tennessee 38105, USA) or human lung cancer H157 cells (obtained from ATCC, Manassas, VA) using Lipofectamine ™ 2000 (Invitrogen). The expression levels of exogenous Bax were compared by Western blot analysis using a Bax antibody.

### Fluorescent immunostaining

For examining the Bax subcellular localization, Bax ^−/−^MEF cells were grown on a 2-well Lab-Tek chamber slide (Nalge Nunc, Naperville, IL) and transfected using 2 µg of plasmid DNA and 5 µl of Lipofectamine 2000. After 16–18 h of incubation, the transfected cells were incubated with 20 ng/ml of a mitochondrion-specific dye (Mitotracker red CMXRos; Molecular Probes Inc., Eugene, OR) for 30 minutes, then fixed and permeablized with methanol and acetone (1∶1) for 5 minutes, and then blocked with 10% normal goat serum for 20 minutes at room temperature. A T7 mouse monoclonal antibody with dilution of 1∶2000 was added to incubate with cells at RT for 90 minutes. After washing, cells were incubated with Alexa Fluor 488 conjugated goat anti-mouse secondary antibody for 40 minutes. Cells were washed with 1x PBS at least four times and observed under a fluorescent microscope (Zeiss). Pictures were taken and colored with the same exposure setting for each experiment.

### Subcellular fractionation

Cells (2×10^7^) were washed with cold 1× PBS and resuspended in isotonic mitochondrial buffer (210 mM mannitol, 70 mM sucrose, 1 mM EGTA, 10 mM Hepes, pH 7.5) containing protease inhibitor mixture set I (Calbiochem), homogenized with a Polytron homogenizer operating for four bursts of 10 s each at a setting of 5, and then centrifuged at 2000×*g* for 3 min to pellet the nuclei and unbroken cells. The supernatant was centrifuged at 13,000×*g* for 10 min to pellet mitochondria as described previously [26]. The second supernatant was further centrifuged at 150,000×*g* to pellet light membranes. The resulting supernatant is the cytosolic fraction. Mitochondria were washed with mitochondrial buffer twice, resuspended in 1% Nonidet P-40 lysis buffer, rocked for 60 min, and then centrifuged at 17,530×*g* for 10 min at 4°C. The resulting supernatant containing mitochondrial proteins was collected. Protein (100 µg) from each fraction was subjected to SDS-PAGE. The purity of each fraction was confirmed by assessing localization of the mitochondria-specific protein prohibitin [30].

### Immunoprecipitation

To test Bax conformational change, Bax^−/−^ MEF cells expressing WT and each of various Bax mutants were lysed using lysis buffer containing CHAPS (150 mM NaCl, 10 mM Hepes pH 7.5, 1% CHAPS). The cell lysate was incubated with 2 µg of 6A7 antibody (BD Pharmingen, San Diego, CA) and 20 µl protein G agarose beads overnight at 4°C. After washing, the resulting beads were boiled with SDS loading sample buffer and subjected to SDS-PAGE for Western blot analysis.

### Alkali extraction to detect Bax insertion

Bax ^−/−^ MEF cells were transfected using pCIneo empty vector, WT and each of Bax mutants. The isolated mitochondria were resuspended in freshly prepared 0.1 M Na_2_CO_3_, pH 11.5, and incubated on ice for 30 min. The samples were then centrifuged at 200,000×*g* for 30 min, and the alkali-resistant pellet was resuspended with 1% Nonidet P-40 lysis buffer, rocked for 60 min, and then centrifuged at 17,530×*g* for 10 min at 4°C. The supernatant containing the nonextractable mitochondrial proteins was collected and subjected to SDS-PAGE. The alkali-resistant Bax (*i.e.* nonextractable or integral) was determined by Western blot using a Bax antibody as described previously [31]–[32].

### Measurement of Cyt c release

To assess cytochrome C release, mitochrondrial and cytosolic fraction were obtained by use of a digitonin-based subcellular fractionation as described [33]–[34]. Equal amount protein of mitochrondial and cytosolic fractions was subjected to SDS-PAGE followed by immunoblotting with anti-Cyt c antibody.

### Measurement of the Bax half-life

Cycloheximide half-life assay was performed as described [35]. Briefly, WT and each of *Bax* mutants were transfected into Bax^−/−^ MEF cells. After 18 h, cyclohexamide (100 µg/ml) was added 5 min prior to starting the indicated time course to block new protein synthesis. Cells were collected and lysed at the indicated times. Bax in total cell lysate was analyzed by Western blot using Bax antibody. To more accurately quantify the turnover rate of Bax, a classical ^35^S-methionine pulse–chase method was also employed. Cells expressing WT and each of Bax mutants were metabolically labeled with ^35^S-methionine for 60 min. The ^35^S-methionine-labeled cells were washed and incubated in fresh methionine-replete RPMI medium 1640 for various time points up to 12 h. ^35^S-labeled Bax was immunoprecipitated by using Bax antibody. The samples were subjected to SDS/10–20% PAGE. The half-life (*t*_1/2_) of Bax was determined by electronic autoradiography as described [22], [36].

### Cell viability assay

After transfection of WT or each of Bax mutants into Bax^−/−^ MEF or H157 cells using LipofectAMINE™ 2000 (Invitrogen), apoptotic and viable cells were detected using an ApoAlert Annexin-V kit (Clontech) according to the manufacturer's instructions. The percentage of annexin-V^low^ (*i.e*. viable) or annexin-V^high^ (*i.e*. apoptotic) cells was determined using the data obtained by fluorescence-activated cell sorter analysis as described [36]–[37]. Additionally, cell viability was also analyzed by luciferase assay as described [38]–[39]. The mammalian expression vector pGL3 (Promega) carrying the firefly luciferase (Luc) gene was co-transfected with WT or each of Bax mutants. A molar ratio of 1∶4 (pGL3: Bax plasmid) was used to co-transfect in Bax^−/−^ MEF or H157 cells in 6-well tissue culture plates. After 48 h, cells were harvested and processed using the luciferase assay system (Promega). Luciferase activity was measured by liquid scintillation counting (Monolight 3010, PharMingen) using 20 µl of the cellular extract. In every experiment, each construct was tested in triplicate, and experiments were repeated three times. Cell viability is shown as the relative luciferase activity of the tested plasmids compared to the vector-only control as described [38]–[39].

### Measurement of mitochondrial membrane potential

Mitochondrial membrane potential (ΔΨ) was measured using an ΔΨ assay kit (Cell Technology, INC) according to the manufacturer's instruction. Briefly, Bax^−/−^ MEF cells in 6-well plates were transiently transfected with empty pCIneo vector, WT or each of Bax mutants for 16 hours. 10 µl of 30X mitochondrial membrane potential dye was added to each well. After incubation for 30 min, cells were trypsinized, washed with 1× wash buffer and analyzed by flow cytometry.

## Results

### The effects of mono- and double-site phosphorylation of Bax on its proapoptotic function

**Figure 1:**
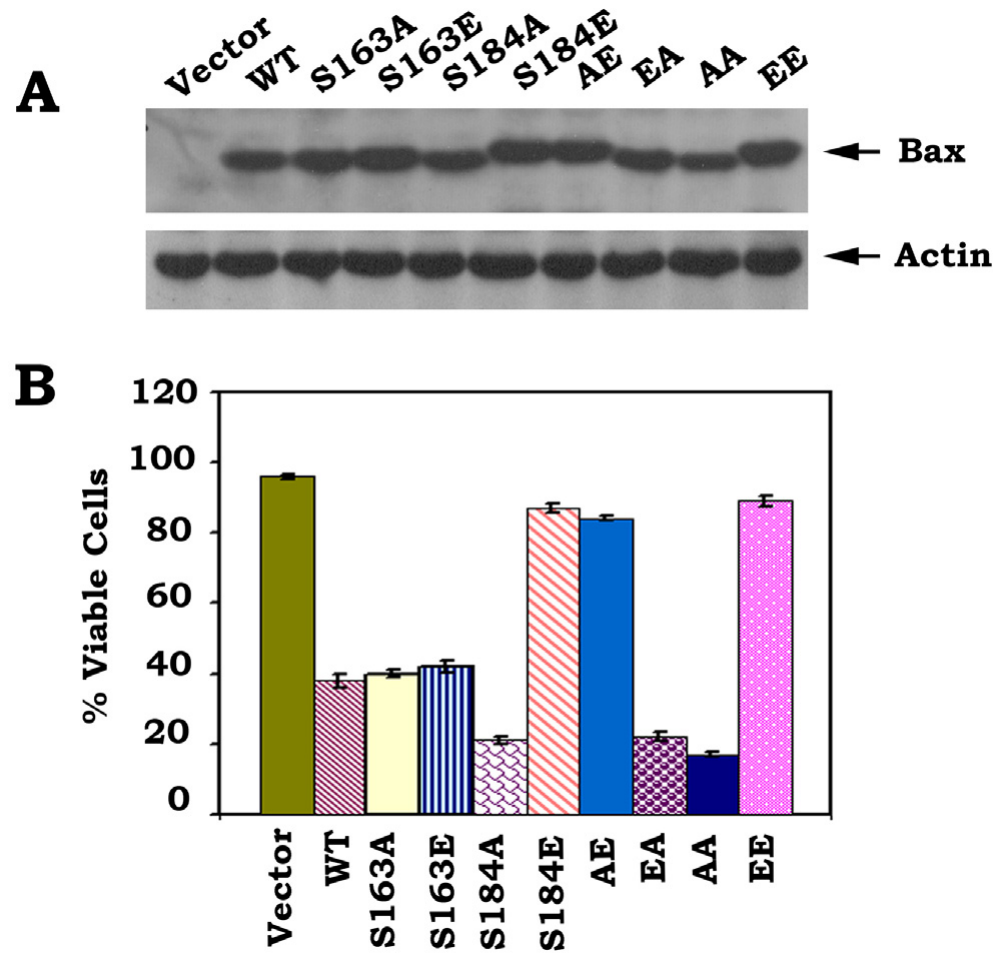
Mono-or double-site Bax phosphorylation regulates its proapoptotic activity. *A*, The pCIneo empty vector, T7-tagged WT and each of A- or E-Bax mutant cDNAs were transfected in Bax^−/−^ MEF cells. After 24 h, expression levels of exogenous Bax protein were determined by Western blot using T7 antibody. *B*, After transfection with WT or each of Bax mutants for 48 h, cell viability was determined by analyzing annexin-V binding on FACS. Data represent the mean ± S.D. of three determinations.

To directly test and compare the function and potency of mono- and double-site phosphorylation, a series of S→A or S→E *Bax* mutants were created to abrogate or mimic, respectively, the charge conferred by phosphorylation (Fig. 1A). After transfection of these *Bax* mutants into Bax^−/−^ MEF cells, the Western blot analysis indicates that introduction of E at the S184 site produces a shifted mobility of Bax band following denaturing electrophoresis. Since only individual S184E, AE and EE but not S163E or EA Bax mutants show this mobility pattern (Fig. 1A), suggesting that the charge conferred by phosphorylation at S184 but not S163 site is essential for inducing this specific conformational change responsible for Bax's altered mobility. We further examined the proapoptotic activities of WT and each of various Bax mutants using an ApoAlert Annexin-V kit as described [36]–[37]. Interestingly, the mutant S184A has more apoptotic activity than WT Bax, and S184E mutant Bax has no significant apoptotic effect on cells. The mutant S163A has no significant difference in apoptotic activity as compared to S163E or WT Bax. The mutants S184E, AE and EE lost their death activities because viabilities of cells expressing these mutants are close to vector-only control. The mutant EA and AA had similar levels of proapoptotic activity as S184A Bax mutant. The ordering of proapoptotic activity for the mutants as follow: S184A≈EA≈AA>WT≈S163A ≈S163E>S184E≈AE≈EE (Fig. 1B). Our findings suggest that the S184 perhaps not the S163 is a critical phosphorylation site in regulating the proapoptotic activity of Bax. Phosphorylation at S184 site causes a conformational change (*i.e*. mobility shift) which may inactivate Bax's proapoptotic function. To rule out a possible cell type or species-specific effect for E- or A-Bax mutants, similar studies were performed in human H157 epithelial lung cancer cells using luciferase assay for evaluating cell viability as described [38]–[39]. This assay is based on the reduction in expression of a luciferase reporter gene that was co-expressed with WT or each of Bax mutants. Overexpression of the proapoptotic Bax is supposed to reduce the luciferase activity in cells. Consistently, similar results were obtained in H157 cells (Fig. S1). This confirms a functional role for Bax phosphorylation in regulating apoptosis that is not limited to a specific cell type or species.

### Phosphorylation of Bax at S163 and S184 distinctly regulates its subcellular localization

Bax translocation from cytosol to mitochondria is an essential event for Bax-mediated intrinsic apoptosis pathway. Cytosolic Bax protein becomes mitochondria-bound in cells undergoing apoptosis [1]. It was reported that C-terminus of Bax plays a critical role in regulation of Bax subcellular localization, deletion of 10 or even only five amino acids from the hydrophobic tail that consists of a total of 21-amino acids could prevent Bax mitochondrial membrane insertion and abrogate its proapoptotic activity [2].

**Figure 2:**
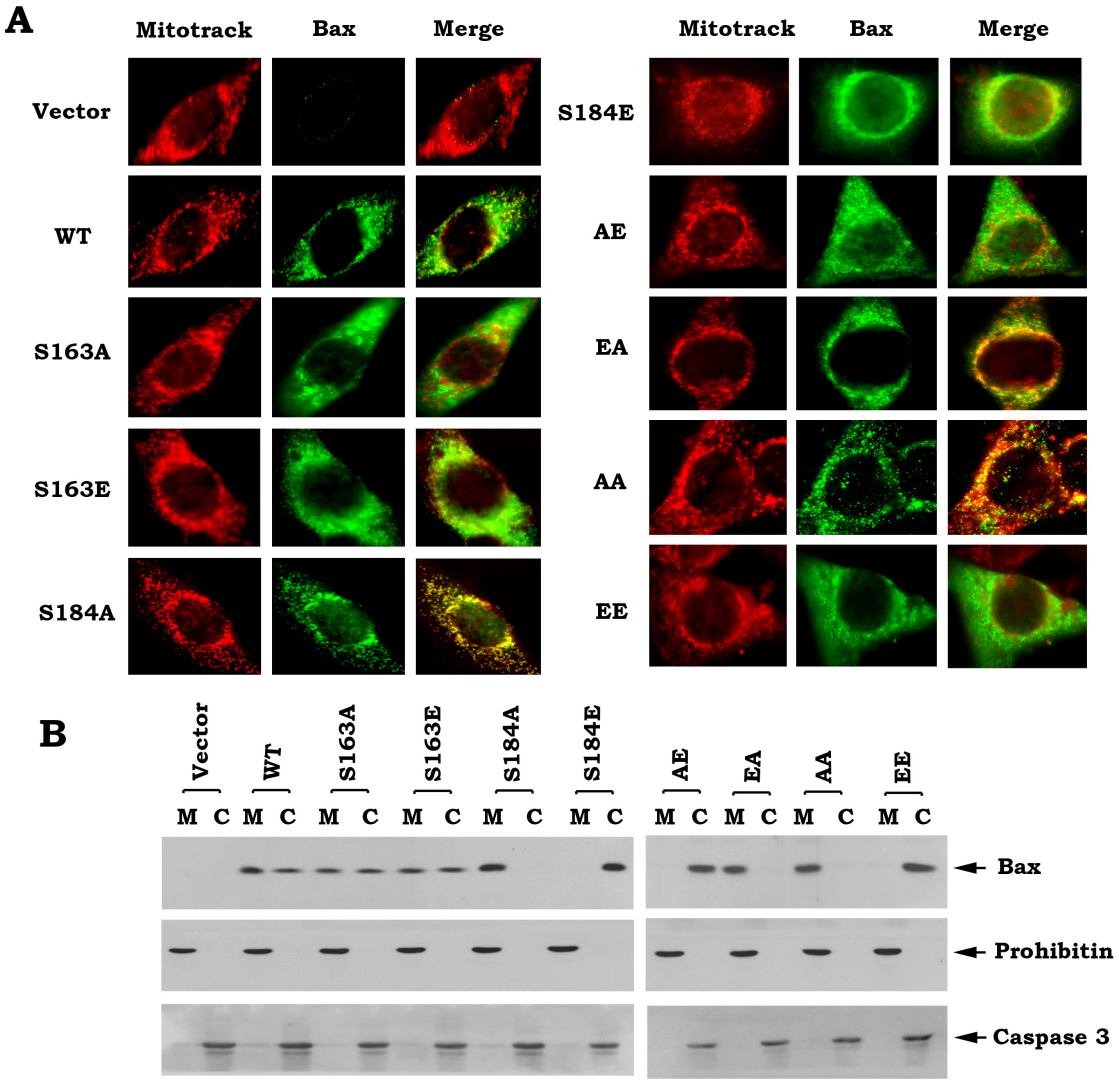
Effect of Bax phosphorylation on its subcellular distribution. *A*, After transfection of WT or each of Bax mutants in Bax^−/−^ MEF cells, cells were incubated with Mitotracker CMXRos (red). After fixation, cells were stained with T7 antibody and Alex 488 conjugated secondary antibody and observed under a fluorescent microscope. *B*, Subcellular fractionation was performed to isolate mitochondrial and cytosolic fractions from Bax^−/−^ MEF cells expressing exogenous WT or each of Bax mutants. Bax protein was analyzed by Western blot using a T7 antibody. The purity for each fraction was monitored by analyzing the fraction-specific proteins (*i.e*. prohibitin, a mitochondrial marker; caspase 3, a specific cytosolic protein).

To uncover the mechanism(s) by which Bax phosphorylation regulates its proapoptotic function, subcellular distribution of WT and each of Bax mutants were examined by immunofluorescent staining and subcellular fractionation analyses. After transient transfection of WT and each of *Bax* mutants in Bax^−/−^ MEF cells, mitotracker and immunofluorescent double stainings indicate that S184A, EA and AA Bax mutants are more co-localized with mitotracker red CMXRos since their emerged images represent stronger yellow color (Fig. 2A). Additionally, S184A and AA Bax mutants represent punctuate, mitochondrial localization. In contrast, S184E and EE Bax mutants have diffuse, cytoplasmic localization (Fig. 2A). WT and other Bax mutants have mixed localizations (*i.e*. diffuse plus punctuate). Consistently, subcellular fractionation analysis reveals that WT, S163A and S163E are localized in both cytosol and mitochondria. S184A, EA and AA Bax mutant proteins are completely localized in mitochondrial membranes while S184E, AE and EE are totally localized in cytosol (Fig. 2B), which support the findings from immunofluorescent staining. To confirm the purity of the subcellular fractions obtained, fraction-specific proteins were assessed by probing the same filters. Prohibitin, an exclusively mitochondrial protein [30], was detected only in the mitochondrial fraction whereas caspase 3, which is a cytosolic protease in growing cells [40], was detected exclusively in the cytosol (Fig. 2B). These results indicate that the mitochondrial and cytosolic fractions are pure without the artifactual cross-contamination.

### Effect of Bax phosphorylation on its 6A7 epitope conformational change

**Figure 3:**
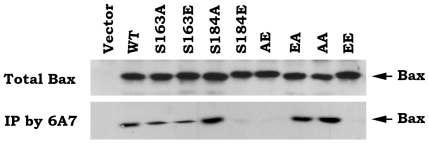
Effect of Bax phosphorylation on its 6A7 epitope conformational change. Immunoprecipitation was performed using 6A7 antibody in total lysates isolated from Bax^−/−^ MEF cells expressing exogenous WT or each of Bax mutants. The 6A7 epitope conformationally changed Bax was analyzed by Western blot using a Bax antibody.

To determine whether the phosphorylation status of Bax affects its active conformational change, an anti-6A7 Bax antibody that specifically measures the conformational change of Bax was used. The monoclonal antibody 6A7, raised against the peptide amino acids 13–19 in the N terminus of Bax, is not able to bind the soluble form of Bax in healthy cells but can recognize Bax after the conformational change associated with membrane insertion occurs in apoptotic cells [41]–[42]. Recent studies further demonstrated that the 6A7 Bax antibody only recognizes conformationally changed, active Bax [27]. Immunoprecipitation experiment using 6A7 antibody reveals that S184A, EA and AA mutants have more abilities to bind to 6A7 antibody as compared to WT, S163A and S163E Bax mutants. Intriguingly, S184E, AE and EE lack abilities to bind to 6A7 antibody (Fig. 3). These findings suggest that the S184 nonphosphorylatable Bax or dephosphorylation of Bax at S184 site may cause a conformational change by exposure of 6A7 epitope that leads to activation of the proapoptotic function of Bax, which is functionally opposite with the mobility shift derived from the phosphomimetic mutation at this site (*i.e*.S184E; Fig. 1 vs. Fig. 3).

### Phosphorylation of Bax regulates its ability to insert into mitochondrial membrane and induce cytochrome c release

**Figure 4:**
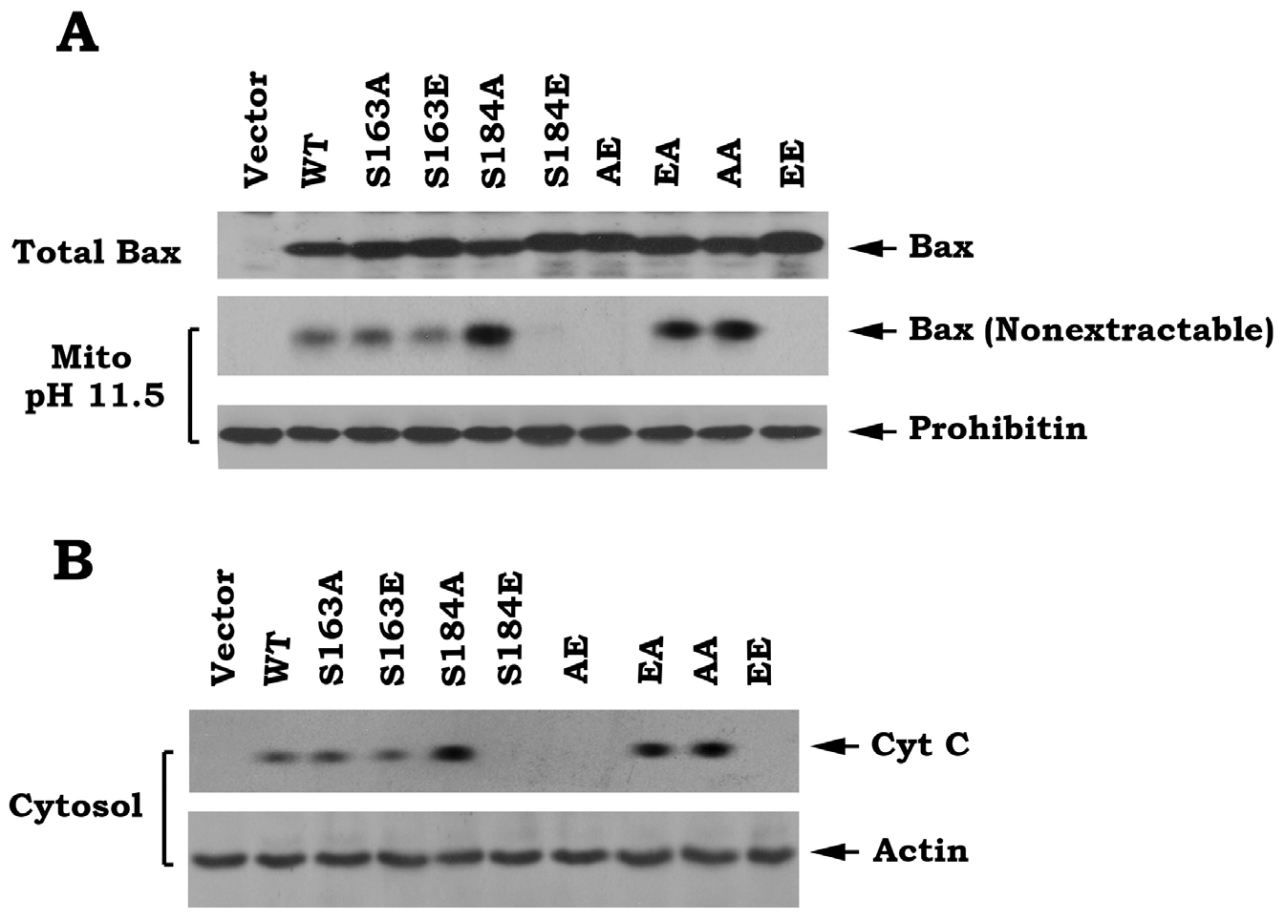
Role of Bax phosphorylation in regulating its abilities to insert mitochondrial membranes and induce Cyt c release. *A*, Mitochondria were isolated from Bax^−/−^ MEF cells expressing exogenous T7-tagged WT or each of Bax mutants and alkali extraction of Bax was performed as described under “Experimental Procedures.” The alkali-resistant Bax (*i.e.* nonextractable) was determined by Western blot using a T7 antibody. Prohibitin, an inner mitochondrial membrane protein, was used as control. *B*, Subcellular fractionation was performed to isolate mitochondria and cytosol. The release of Cyt c from mitochondria into cytosol was detected by Western Blot using Cyt c antibody.

In health cells, Bax is primarily located at the cytosol where it may be loosely associated with the outer mitochondrial membrane. Upon induction of apoptosis, Bax is translocated to mitochondria and becomes integrally associated with the outer mitochondrial membrane [3]. It has been demonstrated that an alkali extraction approach can distinguish whether Bax protein associates with or inserts into mitochondrial membranes [31]. An alkali extraction experiment was employed to test whether Bax phosphorylation at different sites regulates its capacity to insert into mitochondrial membranes. Mitochondria were isolated from Bax^−/−^ MEF cells that express exogenous WT and each of Bax mutants and incubated in 0.1 M Na_2_CO_3_ (pH 11.5) on ice for 30 min and centrifuged at 200,000×g to yield a mitochondrial pellet. The resulting alkali extracted mitochondrial membrane pellet will be resuspended in 1% NP-40 lysis buffer. The alkali-resistant, integral Bax will be analyzed by Western blot. Results reveal that S184A, EA and AA mutants display more Bax proteins resistant to alkali extraction as compared to WT, S163A and S163E mutants (Fig. 4A). In contrast, S184E, AE and EE mutant proteins could not be observed in mitochondrial membranes after alkali treatment (Fig. 4A). These findings reveal that S184A, EA and AA mutant Bax proteins have more ability to insert into mitochondrial membranes and that S184E, AE and EE mutant proteins lost this capacity. To further test whether the mitochondrially inserted Bax promotes Cyt c release, subcellular fractionation experiments were carried out in cells expressing WT or each of A- or E-Bax mutants. Consistently, S184A, EA and AA Bax mutants more potently induce Cyt c release as compared to WT, S163A and S163E mutants (Fig. 4B). In contrast, S184E, AE and EE lost their abilities to induce Cyt c release (Fig. 4B).

### Phosphorylation of Bax reduces its stability

**Figure 5:**
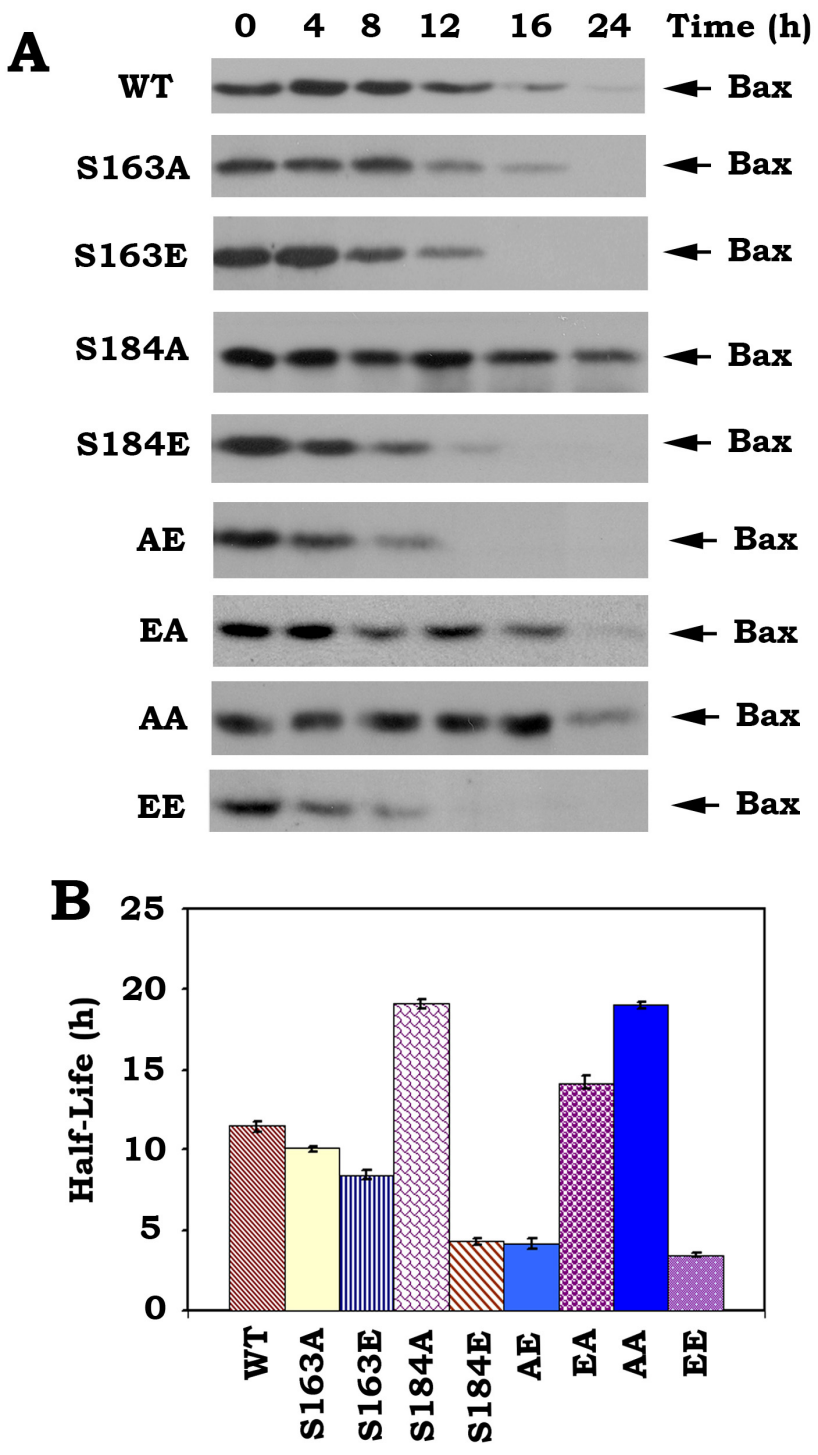
Effect of Bax phosphorylation status on its half-life in cells. *A*, Bax^−/−^ MEF cells expressing exogenous WT or each of Bax mutants were treated with 100 µg/ml cycloheximide 5 min prior to starting the indicated time course. Bax was analyzed by Western blot. *B*, Bax^−/−^ MEF cells expressing exogenous WT or each of Bax mutants were metabolically labeled with ^35^S-methionine. The half-life of Bax was determined by classic pulse-chase analysis. Data represent the mean ± S.D. of three determinations.

It has been demonstrated that Bax degradation occurs through the ubiquitin/proteasome-dependent pathway and inhibition of Bax degradation enhances the proapoptotic function of Bax [43]. To test how Bax phosphorylation at S163 and S184 affects its stability, a cycloheximide half-life (t_1/2_) assay was performed in cells expressing WT or A- or E-containing Bax mutants as described [35]. To more accurately evaluate the turnover rate of WT or A- or E-containing Bax mutants, we also used the classical ^35^S-methionine pulse–chase method to quantify the half-life (t_1/2_) of Bax by electronic autoradiography as we previously described [22], [36]. The t_1/2_ of S184E, AE and EE was found to be significantly shorter (*i.e*.3.5∼4.3 h) than that of WT Bax (*i.e*. 11.5 h). In contrast, S184A, EA and AA have a significant longer half-life (*i.e*. 14.2∼19.1 h). The t_1/2_ of S163A or S163E is shorter than WT but longer than S184E (Fig. 5). These results indicate that the charge conferred by phosphorylation at S184 significantly reduces Bax's stability to turnover. Therefore, we propose that placing a charge equivalent to a phosphate at the S184 phosphorylation site in the C-terminus will, in addition to directly inactivating Bax's proapoptotic function, also promote its proteolytic degradation that lead to cell survival (Figs 1 and 5). The nonphosphorylatable S184A containing Bax mutants have enhanced stability to proteolysis, which may further enhance the proapoptotic function of Bax.

### Effect of Bax phosphorylation on mitochondrial membrane potential (ΔΨ)

**Figure 6:**
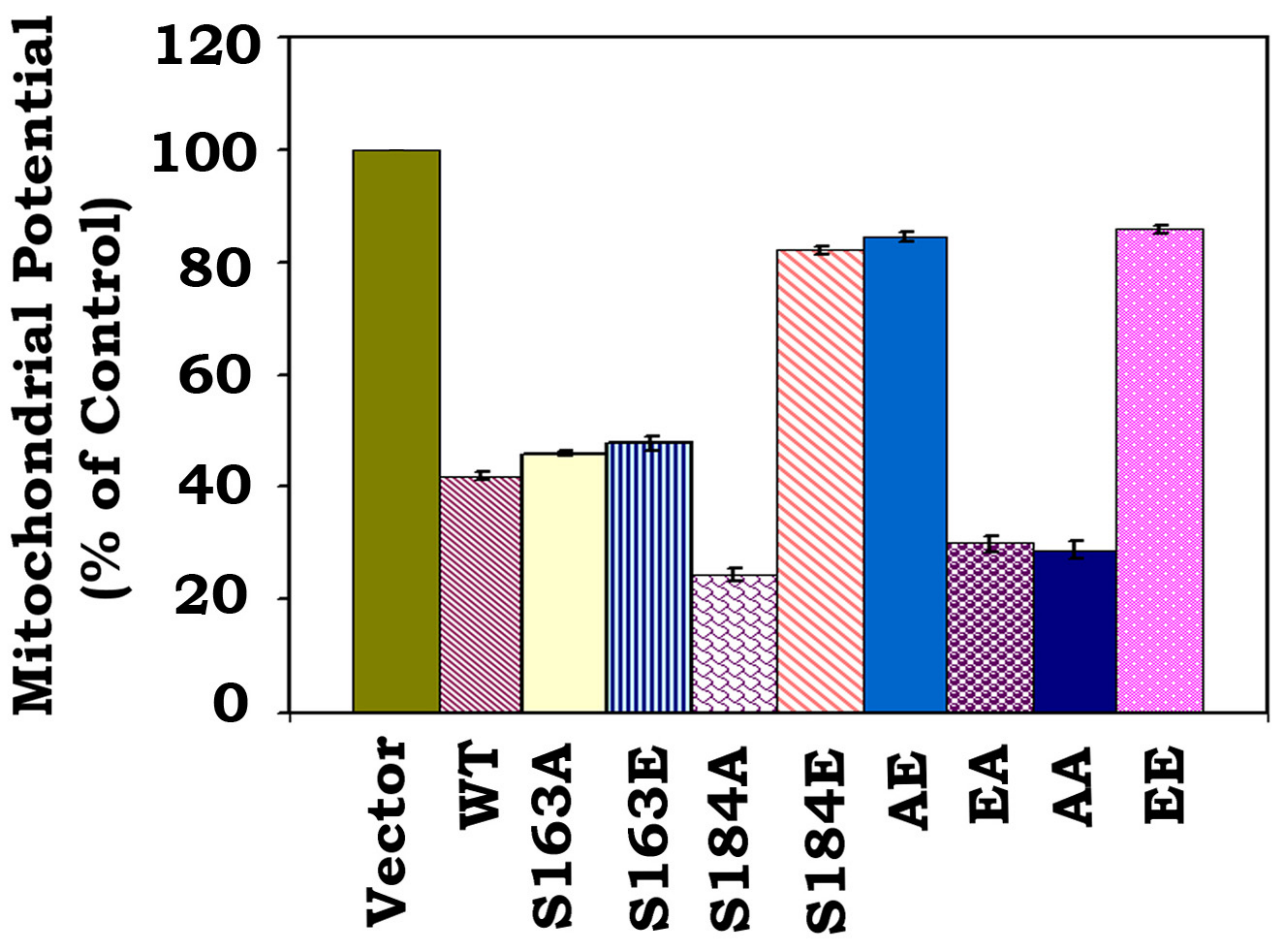
Effect of Bax phosphorylation on mitochondrial membrane potential. The pCIneo empty vector, WT or each of Bax mutants were transfected into Bax^−/−^ MEF cells. After 16 h, cells were stained with cationic dye and analyzed by FACS. Data represent the mean ± S.D. of three determinations.

The mitochondrial permeability transition is an important step in the induction of cellular apoptosis. During this process, the electrochemical gradient across the mitochondrial membrane collapses. This collapse may occur through the formation of pores in the mitochondria by activated, dimerized Bax [44]. After transfection of WT or each of Bax mutants, ΔΨ was analyzed as described [45]. The cationic dye is cell permeable and has strong fluoresecent signal and exhibits low membrane potential independent binding and toxicity. In healthy cells, cationic dye is accumulated by the mitochondria in proportion to membrane potential. In apoptotic cells, where ΔΨ is compromised, the cationic dye does not accumulate in the mitochondria and these cells exhibit a lower fluorescence signal. Consistently, expression of S184A, EA or AA mutant Bax causes more ΔΨ loss as compared to WT, S163A and S163E mutants. In contrast, expression of S184E, AE or EE mutant Bax did not significantly lose ΔΨ (Fig. 6).

## Discussion

The Bcl2 family lies at the central of the intrinsic pathway to apoptosis, the interactions between the family members being critical for determining the fate of a cell (*i.e*. alive or dead) [46]–[47]. The multi-domain pro-apoptotic members, including Bax and Bak, have been demonstrated that one or other of these proteins are required for apoptotic cell death through the release of apoptogens from mitochondria [6], [48]. However, the mechanism by which the cell death mediator Bax becomes activated to cause mitochondrial damage remains elusive. Mounting evidence indicates that the proapoptotic function of Bax can be regulated by phosphorylation at various sites through activation of various protein kinases, including PI3K/AKT [25]–[26], GSK-3β [28], JNK and p38 kinases [49]. Intriguingly, phosphorylation at S163 and S184 has been reported to play an inverse role in regulating the proapoptotic function of Bax [25]–[26], [28]. To flesh out the functional contribution of individual phosphorylation site(s), we chose to abrogate Bax phosphorylation by introducing a conserved, non-phosphorylated alanine (A) at the S163 or S184 phosphorylation site(s). Similar mutant(s) carrying a glutamate (E) at these same sites were prepared in order to functionally mimic the charge conferred by the phosphate (*i.e*. phosphomimetic). The power of this genetic approach is intensified in these studies because a direct comparison can be made between the non-phosphorylatable (*i.e*. S→A) and phosphomimetic (*i.e*. S→E) Bax mutants expressed at the same levels in the same cell types. We believe that this comparative approach can provide a definitive answer to explain how, and at which site(s) phosphorylation affects the proapoptotic function of Bax.

Results clearly indicate that the non-phosphorylatable S184A-containing Bax mutants (*i.e*. S184A, EA and AA) function as active forms of Bax, which exhibit the highest cell killing activity. Inversely, the phosphomimetic S184E-containing mutants (*i.e*. S184E, AE and EE) almost lost their proapoptotic activities (Figs. 1 and S1). These findings support and extend our and others' previous studies that phosphorylation of Bax at S184 can inactivate the proapoptotic function of Bax and that dephosphorylation of Bax at this site renders Bax more potent killing capacity [25]–[27]. However, levels of the killing activity of the non-phosphorylatable S163A and the phosphomimetic S163E have no significant difference as compared to WT Bax (Fig. 1). This suggests that phosphorylation or dephosphorylation of Bax at S163 site may not significantly alter its proapoptotic function. These results are difficult to reconcile with previous report that GSK-3β-induced Bax phosphorylation at S163 activates its proapoptotic function in neuronal cells [28]. While the reason for the discrepancy is not completely clear, it is possible to result from a cell-type specific effect. Since results reported here are obtained by direct comparison of the Bax mutants that either carry charge that mimics phosphorylation or a mutation unable to accept a phosphate charge, we propose that S184 but not S163 is the major Bax phosphorylation site that can regulate the proapoptotic function of Bax by manipulating the phosphorylation status at this site.

A conformational change conferred by exposure of 6A7 epitope in the N-terminus (amino acids 13–19) has been demonstrated to activate the proapoptotic function of Bax [27], [41]–[42]. Here our findings reveal that destruction of the S184 phosphorylation site by alanine substitution (*i.e*. S→A) can cause such a conformational change (*i.e*. exposure of 6A7 epitope) because the S184A containing Bax mutants (*i.e*. S184A, EA and AA) have more abilities to bind to 6A7 antibody (Fig. 3). Importantly, these Bax mutants with the 6A7 epitope conformational change function as active forms of Bax that more efficiently insert into mitochondrial membranes leading to Cyt c release and apoptosis (Figs 1 and 4). Interestingly, mimicking phosphorylation at S184 site by a glutamate substitution (*i.e*. S→E) in the C-terminal tail of Bax results in another type of conformational change that represents a mobility-shift (Fig. 1A) but the functional significance of this conformational change remains unclear. Because the S184E-containing Bax mutants (*i.e*. S184E, AE and EE) with mobility-shift lost their abilities to target and insert into mitochondrial membranes, which eventually fails to induce Cyt c release from mitochondria (Figs 2 and 4). Additionally, this conformational change with mobility-shift renders Bax more sensitive to degradation (Fig. 5). Based on these important findings, we propose that the conformational change conferred by S184 site phosphorylation may directly inactivate the proapoptotic function of Bax. Since the conformational change(s) and the proapoptotic activity of Bax are dependent on the S184 but not the S163 phosphorylation site, manipulation of the phosphorylation status at S184 should represent novel strategies for treatment of cancer by altering the apoptotic activity of Bax in tumor cells.

## Supporting Information

Figure S1 Mono-or double-site Bax phosphorylation regulates its proapoptotic activity in H157 cells.
(0.09 MB PDF)
