# Supplementary material for: Mono- or Double-Site Phosphorylation Distinctly Regulates the Proapoptotic Function of Bax
Source: PLoS One. 2010 Oct 14;5(10):e13393. doi: 10.1371/journal.pone.0013393 (PMC2954808; doi:10.1371/journal.pone.0013393)
Supplement: Figure S1 — Mono-or double-site Bax phosphorylation regulates its proapoptotic activity in H157 cells. (0.09 MB PDF) [file pone.0013393.s001.pdf]

Supplemental Data

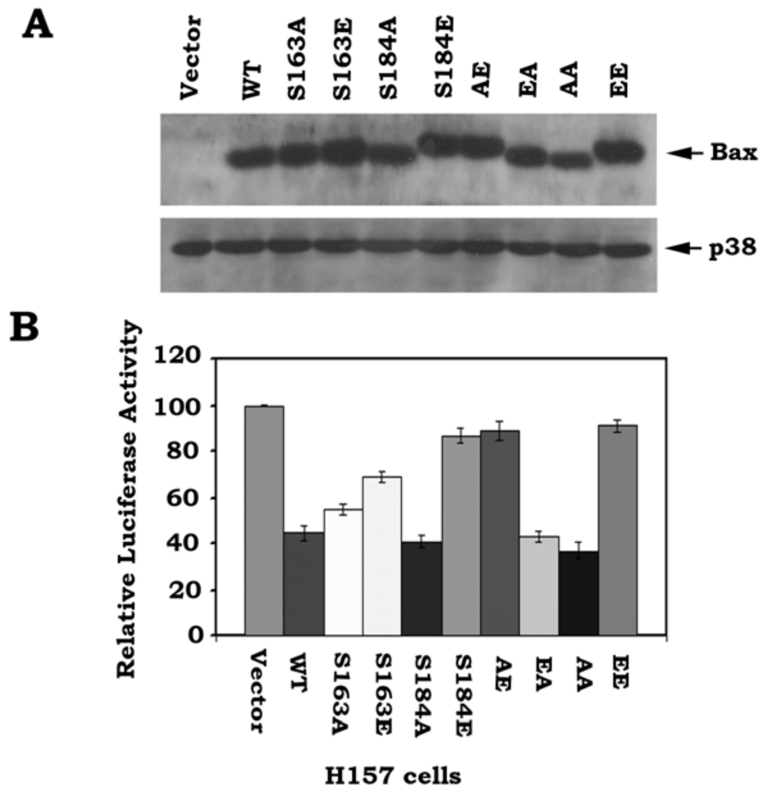

Figure S1. Mono-or double-site Bax phosphorylation regulates its proapoptotic activity in H157 cells. *A*, The pCIneo empty vector, T7-tagged WT and each of A- or E-Bax mutant cDNAs were transfected in H157 cells. After 24h, levels of exogenous Bax protein were determined by Western blot using T7 antibody. *B*, The mammalian expression vector pGL3 (Promega) carrying the firefly luciferase (Luc) gene was co-transfected with WT or each of Bax mutants in H157 cells. After 48h, cell viability was determined by luciferase assay as described in “Methods”. Data represent the mean ± S.D. of three determinations.
